# Pharmacodynamic modeling of cardiac biomarkers in breast cancer patients treated with anthracycline and trastuzumab regimens

**DOI:** 10.1007/s10928-018-9579-8

**Published:** 2018-02-10

**Authors:** Aurelia H. M. de Vries Schultink, Annelies H. Boekhout, Jourik A. Gietema, Artur M. Burylo, Thomas P. C. Dorlo, J. G. Coen van Hasselt, Jan H. M. Schellens, Alwin D. R. Huitema

**Affiliations:** 1 Department of Pharmacy & Pharmacology, Antoni van Leeuwenhoek – The Netherlands Cancer Institute, Louwesweg 6, 1066 EC Amsterdam, The Netherlands; 2 Division of Pharmacology, Antoni van Leeuwenhoek – The Netherlands Cancer Institute, P.O. Box 90203, 1006 BE Amsterdam, The Netherlands; 3 Department of Medical Oncology, University Medical Center Groningen, University of Groningen, Groningen, The Netherlands; 4 Division of Systems Biomedicine and Pharmacology, Leiden Academic Centre for Drug Research, Leiden University, Einsteinweg 55, 2333 CC Leiden, The Netherlands; 5 Division of Pharmacoepidemiology & Clinical Pharmacology, Science Faculty, Utrecht Institute for Pharmaceutical Sciences (UIPS), Utrecht University, P.O. Box 80082, 3508 TB Utrecht, The Netherlands; 6 Department of Clinical Pharmacy, University Medical Center Utrecht, Utrecht University, P.O. Box 85500, 3508 GA Utrecht, The Netherlands

**Keywords:** Trastuzumab, Anthracyclines, Cardiac biomarkers, Breast cancer, Pharmacodynamics, Pharmacometrics

## Abstract

Trastuzumab is associated with cardiotoxicity, manifesting as a decrease of the left-ventricular ejection fraction (LVEF). Administration of anthracyclines prior to trastuzumab increases risk of cardiotoxicity. High-sensitive troponin T and N-terminal-pro-brain natriuretic peptide (NT-proBNP) are molecular markers that may allow earlier detection of drug-induced cardiotoxicity. In this analysis we aimed to quantify the kinetics and exposure–response relationships of LVEF, troponin T and NT-proBNP measurements, in patients receiving anthracycline and trastuzumab. Repeated measurements of LVEF, troponin T and NT-proBNP and dosing records of anthracyclines and trastuzumab were available from a previously published clinical trial. This trial included 206 evaluable patients with early breast cancer. Exposure to anthracycline and trastuzumab was simulated based on available dosing records and by using a kinetic-pharmacodynamic (K-PD) and a fixed pharmacokinetic (PK) model from literature, respectively. The change from baseline troponin T was described with a direct effect model, affected by simulated anthracycline concentrations, representing myocyte damage. The relationship between trastuzumab and LVEF was described by an indirect effect compartment model. The EC_50_ for LVEF decline was significantly affected by the maximum troponin T concentration after anthracycline treatment, explaining 15.1% of inter-individual variability. In this cohort, NT-proBNP changes could not be demonstrated to be related to anthracycline or trastuzumab treatment. Pharmacodynamic models for troponin T and LVEF were successfully developed, identifying maximum troponin T concentration after anthracycline treatment as a significant determinant for trastuzumab-induced LVEF decline. These models can help identify patients at risk of drug-induced cardiotoxicity and optimize cardiac monitoring strategies.

**Electronic supplementary material:**

The online version of this article (10.1007/s10928-018-9579-8) contains supplementary material, which is available to authorized users.

## Introduction

Trastuzumab is a monoclonal antibody that targets the human epidermal growth factor receptor 2 (HER2) and is used to treat HER2-positive metastatic and early breast cancer and metastatic gastric cancer [1–3]. Despite improvement in overall and progression free survival, application of the drug is hampered by cardiac adverse effects, leading to dose reductions, dose-delays and treatment interruption or withdrawal with an increased recurrence risk as a consequence [4]. Trastuzumab-induced cardiotoxicity is manifested as an asymptomatic decrease of the left-ventricular ejection fraction (LVEF) and development of congestive heart failure [5]. The mechanism behind this cardio-toxic effect is not completely elucidated, though it has been demonstrated that trastuzumab causes structural and functional changes to contractile proteins in the heart muscle [6, 7]. These changes rarely lead to cell death, that might explain why the decrease in LVEF values is partly reversible when trastuzumab treatment is discontinued [5]. In addition, patients treated with trastuzumab are often pretreated with anthracyclines. Anthracyclines can irreversibly damage myocytes, possibly by generation of reactive oxygen species and lipid peroxidation of the cell membrane of cardiomyocytes [8]. A cumulative lifetime dose exceeding 550 mg/m^2^ for doxorubicin and 950 mg/m^2^ for epirubicin has been associated with increased incidence of heart failure [9, 10]. These cumulative dose thresholds are therefore clinically applied to limit the risk for cardiac damage. Since both trastuzumab and anthracyclines can lead to cardiac dysfunction, it is not surprising that a higher incidence of cardiac dysfunction has been reported for patients treated with anthracyclines concomitantly or prior to trastuzumab [11]. However, a high variability in susceptibility to cardiotoxicity is seen for patients treated with anthracyclines and trastuzumab. Cardiac function can be monitored during treatment using echocardiography or multiple gated acquisition (MUGA) scan to determine the LVEF. Attempts have been made to optimize cardiac monitoring strategies, allowing for a better identification of patients that experience cardiotoxicity and decrease the number of LVEF measurements in low-risk patients [12]. Cardiac biomarkers such as troponin T and N-terminal-pro-brain natriuretic peptide (NT-proBNP) are molecular markers suggested to allow earlier detection of drug-induced cardiotoxicity compared to LVEF measurement [13]. Cardiac troponins are indicative of myocyte damage and are suggested to predict patients at risk of cardiotoxicity during trastuzumab treatment who are pretreated with anthracyclines [14]. In addition, elevated baseline concentrations of troponin I and troponin T have been related to an increased risk of LVEF decrease [13]. NT-proBNP is a marker for heart failure. Currently, troponins and NT-proBNP are used as cardiac biomarkers for prognosis and diagnosis of myocardial infarction and heart failure, respectively [15]. However, no evidence exists for anti-cancer drug management based on abnormal cardiac biomarker concentrations. Development of a pharmacokinetic–pharmacodynamic (biomarker) model can give insight in the time course of cardiac biomarkers during treatment and help identify the optimal time point of cardiac biomarker assessment. In this analysis, we aim to quantify the kinetics and exposure–response relationship of LVEF, troponin T and NT-proBNP measurements, in patients with early breast cancer receiving anthracyclines followed by trastuzumab. Ultimately, the quantification of cardiac biomarkers could help identify patients at increased risk of developing cardiotoxicity and optimize clinical management of trastuzumab-induced cardiotoxicity.

## Methods

### Patients and data

The analysis conducted in this study was based upon data from patients with HER2-positive early breast cancer from a previously conducted randomized, placebo-controlled clinical trial, investigating protection for trastuzumab-induced cardiotoxicity with angiotensin II-receptor inhibitor candesartan. All patients received adjuvant treatment with anthracycline-containing chemotherapy, either doxorubicine or epirubicine, followed by trastuzumab treatment for 52 weeks. Patients were randomized to receive candesartan or placebo (1:1) daily, starting from the first trastuzumab administration until 26 weeks after the last trastuzumab administration. The trial demonstrated that concomitant candesartan treatment did not protect against decreases in LVEF during trastuzumab treatment. [16]

Repeated measurements for LVEF were available for all patients. LVEF was determined with MUGA scan or echocardiography. Autologous red blood cells (400 MBq Tc-99m labelled) were injected and acquisition was performed in 6 min with a large-field-of-view gamma camera with a low energy all-purpose parallel-hole collimater. Troponin T and NT-proBNP concentrations were available for 92% of patients and were measured in plasma samples using a sandwich immunoassay (Modular E system, Roche Diagnostics). The lower-limit of quantification (LLOQ) for NT-proBNP was 5 pg/mL and for (high sensitive) troponin T 3 ng/L. Troponin T and NT-proBNP measurements were scheduled at the following visits: before starting anthracycline treatment, at baseline before starting trastuzumab treatment and 3, 12, 24, 36, 52, 64, 78 and 92 after starting trastuzumab treatment. LVEF was evaluated at the same time points, except for the 3 week and 64 week visit since start trastuzumab treatment. Additionally, individual patient dosing records, including time of administration and dosages of anthracycline and trastuzumab were available. None of the patients received a cumulative dose above 550 or 950 mg/m^2^ for doxorubicin and epirubicin, respectively.

### Modeling cardiac biomarkers

#### Structural models

Exposure to anthracyclines and trastuzumab were predicted using individual dosing records of the drugs and simulated using different approaches. A K-PD approach was applied for the anthracyclines (doxorubicin and epirubicin) [17]. The trastuzumab PK profiles were obtained using fixed effect parameters from a previously published PK model for HER2-positive breast cancer patients [18].

Exploratory plots were used to determine pharmacodynamic modeling starting points for the three cardiac biomarkers. The plots demonstrated an increase in troponin T during anthracycline treatment, and a gradual decrease of troponin T during trastuzumab treatment. Therefore, troponin T changes were assumed to be related to anthracycline treatment only. Troponin T samples were taken before anthracycline treatment, approximately 21 days after the last anthracycline dose and during trastuzumab treatment, with limited samples available during anthracycline treatment. Therefore, direct and indirect effect models were evaluated to identify if the troponin T peak concentration occurred right after the last administration of anthracyclines or if the peak was delayed after treatment.

A previously published model by our group was used as a starting point for modeling trastuzumab-induced LVEF decrease [19]. In this model the LVEF decline was described by an effect compartment, demonstrating cardiac damage induced by trastuzumab treatment. The delay in LVEF decline in relation to trastuzumab treatment (the cardiac damage onset rate) was kept equal to the rate of recovery.

NT-proBNP changes were evaluated during anthracycline and trastuzumab treatment separately. A model that described NT-proBNP concentrations to be inversely associated with LVEF values was evaluated. In addition, NT-proBNP baseline values prior to initiation of either anthracycline or trastuzumab treatment were evaluated as covariates.

#### Statistical models

Between subject variability (BSV) was evaluated for all structural model parameters using an exponential error model:

$$P_{i} = P_{pop} \cdot \exp \left( {\eta_{i} } \right)$$

where *P*_*i*_ is the individual parameter estimate for individual *i*, *P*_*pop*_ the population parameter estimate and η_i_ the individual value of between-subject variability for subject *i*, where η_i_ was assumed to be normally distributed with mean 0 and variance ω^2^. Off-diagonal elements of the variance–covariance (omega) matrix, were evaluated to identify covariances between the individual random effects. These covariances were used to derive the correlations between the random-effects. Residual unexplained variability was described as a proportional error model for all cardiac biomarkers:

$$C_{obs,ij} = C_{pred,ij} \cdot \left( {1 + \varepsilon_{p,ij} } \right)$$

where $C_{obs,ij}$ represents the observed concentration for individual *i* and observation *j*, $C_{pred,ij }$ represents the individual predicted concentration, *ɛ*_*p*,*ij*_ the proportional error distributed following N (0,σ^2^).

### Covariate analysis

Different covariates were included in the covariate analysis, based on physiological plausibility and clinical relevance. The following covariates were evaluated for the anthracycline-troponin T model and the trastuzumab-LVEF model: age, hypertension diagnosis and status, radiotherapy of the chest, laterality of radiotherapy and type of anthracycline administered. Additionally, type of anthracycline (epirubicin or doxorubicin) was evaluated as a covariate for the anthracycline-troponin T model, and baseline LVEF values (prior to initiation of trastuzumab) and time between last anthracycline dose and first trastuzumab dose for the trastuzumab-LVEF model. Since patients in this cohort were randomized to receive either placebo or candesartan to prevent or alleviate trastuzumab-induced cardiotoxicity, treatment group was evaluated as a covariate. In addition, the dose normalized cumulative dose of anthracycline and the predicted maximum concentration of troponin T reached by the previous anthracycline treatment were evaluated as a covariates. Binary covariates (previous radiotherapy, hypertension diagnosis, type of anthracycline administered) were implemented using the following equation:

$$P_{i} = P_{pop} \cdot \left( {\theta_{\text{cov}} } \right)^{COV}$$

where *θ*_*cov*_ is the covariate effect parameter and COV the covariate value. Continuous covariates (*COV*_*cont*_) (time between last anthracycline dose and initiation of trastuzumab, maximum concentrations of troponin T) were normalized to the median value of the covariate (*COV*_*median*_) and implemented as follows:

$$P_{i} = P_{pop} \cdot \left( {\frac{{COV_{cont} }}{{COV_{median} }}} \right)^{{\theta_{\text{cov}} }}$$

Categorical covariates (laterality of radiotherapy and hypertension status) were implemented by estimating a separate parameter for each category. The selected physiologically plausible and clinically relevant covariates were evaluated using a forward inclusion and backward elimination method. A significance level of *p* < 0.01 was set for the forward inclusion, corresponding to a decrease of OFV of > 6.63. For backward elimination, a significance level of *p* < 0.005 was set, corresponding to an increase of OFV of > 7.88.

### Model evaluation

Models evaluation was performed using general goodness-of-fit (GOF) plots, plausibility, stability and precision of parameter estimates and change in objection function value (OFV) [20]. A *p* < 0.01 was considered significant, meaning that an OFV drop of > 6.63 for hierarchical models (degree of freedom = 1, Chi squared distribution) was considered as a significant improvement. Since troponin T samples were taken predominantly before anthracycline treatment and approximately 21 days after the last anthracycline dose, predicted troponin T concentrations at day 21 after the last anthracycline administration were also evaluated as a covariate. In addition, the final model for LVEF was evaluated for the placebo and the candesartan group, separately, in order to identify possible differences related to study treatment.

### Software

Data management and graphical evaluation were performed using R (version 3.0.1) [21]. Nonlinear mixed effects modeling was performed using NONMEM (version 7.3.0, ICON Development Solutions, Ellicott City, MD, USA) and Perl-speaks-NONMEM (version 4.4.8) [22, 23]. Piraña (version 2.9.2) was used as graphical user interface [24]. Models were estimated using First Order Conditional Estimation method with η–ε interaction (FOCE-I).

## Results

### Patients and data

In the final analysis, 206 patients were included. A total of 1444 LVEF measurements (96% by MUGA scan) were available for 206 patients with a median [range] of 8 [2–9] measurements per patient. Troponin T and NT-proBNP measurements were available for 190 patients, with a total of 1230 troponin T measurements, 7 [1–11] measurements per patient, and 1028 NT-proBNP measurements, 6 [1–10] measurements per patient. Concentrations below the LLOQ were divided by two in the final dataset (4.6% of the troponin T observations and 2.5% of NT-proBNP were below the LLOQ). Part of the patient characteristics are depicted in Table 1. The clinical data have been extensively described elsewhere [16].

**Table 1:** Patient characteristics

	Median [range]	N
Age at randomization (years)	50 [25–69]	–
Number of anthracycline cycles	4 [2–6]	–
Absolute doses of anthracyclines		
Doxorubicin (mg)	110 [75–150]	–
Epirubicin (mg)	170 [100–200]	–
Number of trastuzumab cycles	23 [5–46]	–
Trastuzumab doses		
3 weekly schedule	8–6 mg/kg	62
Weekly schedule	4–2 mg/kg	144
Time between last anthracycline dose and first trastuzumab dose (days)	21 [14–217]	

	No. of patients (n = 206)	%
Measurements		
LVEF measurements available	206	100
LVEF baseline—before anthracycline	173	84.0
LVEF baseline—before trastuzumab	205	99.5
Troponin T measurements available	190	92.2
NT-proBNP measurements available	190	92.2
Type of anthracycline treatment		
Doxorubicin	181	87.9
Epirubicin	25	12.1
Clinically relevant decline in LVEF^a^		
Yes	37	18
No	169	82
Medical history		
Previous radiotherapy		
Yes	112	45.6
No	94	54.4
Laterality of radiotherapy		
Left	58	28.2
Right	54	26.2
No radiotherapy	94	45.6
Hypertension ever diagnosed		
Yes	24	11.7
No	182	88.3
Hypertension status		
Past	5	2.4
Dormant	12	5.8
Active	7	3.4
No hypertension	182	88.3

^a^A decline in LVEF was assumed clinically relevant if LVEF values decreased with 15% or more from baseline or if a value of < 45% was reached

Data and models for the cardiac biomarkers troponin T, LVEF, and NT-proBNP were explored separately in relation to the anthracycline concentration–time profiles (troponin T and NT-proBNP) and to the trastuzumab concentration–time profiles (LVEF and NT-proBNP). The final model parameter estimates are summarized in Table 2.

**Table 2:** Parameter estimates for cardiac biomarker models anthracycline-troponin T and trastuzumab-LVEF

Parameter	Unit	Parameter estimate	RSE (%)	Shrinkage (%)
Anthracycline—troponin T model				
Troponin T baseline (TRP_0_)	ng/L	4.72	3.5	–
Elimination rate constant K-PD model (*k*_*e*_)	Day^−1^	8.49 × 10^−3^	4.0	–
Proportional effect (anthracyclines-troponin T) (SLOPE)	ng^−1^ L	8.84 × 10^−3^	7.0	–
Proportional anthracycline-type effect on SLOPE		0.524	17.5	–
Between-subject variability (%)				
Slope effect on TRP_0_ (*SLOPE*)	CV	57.7	23.3	31.0
Troponin T baseline (*TRP*_0_)	CV	39.2	9.9	12.6
Residual variability				
Proportional residual error troponin T	%	30.1	4.2	11.2
Trastuzumab—LVEF model				–
LVEF baseline value (LVEF_0_)		0.599	0.6	–
Recovery half-life (*T*_1/2*rec*_)	Day	67.9	17.2	–
Sensitivity to LVEF decline (EC_50_)	mg/L	2.18 × 10^5^	23.4	–
Maximum troponin T effect on EC_50_		− 1.16	23.4	–
Between-subject variability (%)				
LVEF baseline value (LVEF_0_)	CV	7.07	16.7	9.9
Sensitivity to LVEF decline (EC_50_)	CV	82.9	43.1	26.0
Correlation $\omega_{{{\text{LVEF}}_{0} }} \sim \omega_{{{\text{EC}}_{50} }}$^a^	–	0.585		
Residual variability				
Proportional residual error LVEF	%	7.8	2.9	8.3

*CV* coefficient of variation, *SD* standard deviation, *RSE* relative standard error

^a^Correlation derived from the variance–covariance matrix of the random effects

### Troponin T

**Fig. 1:**
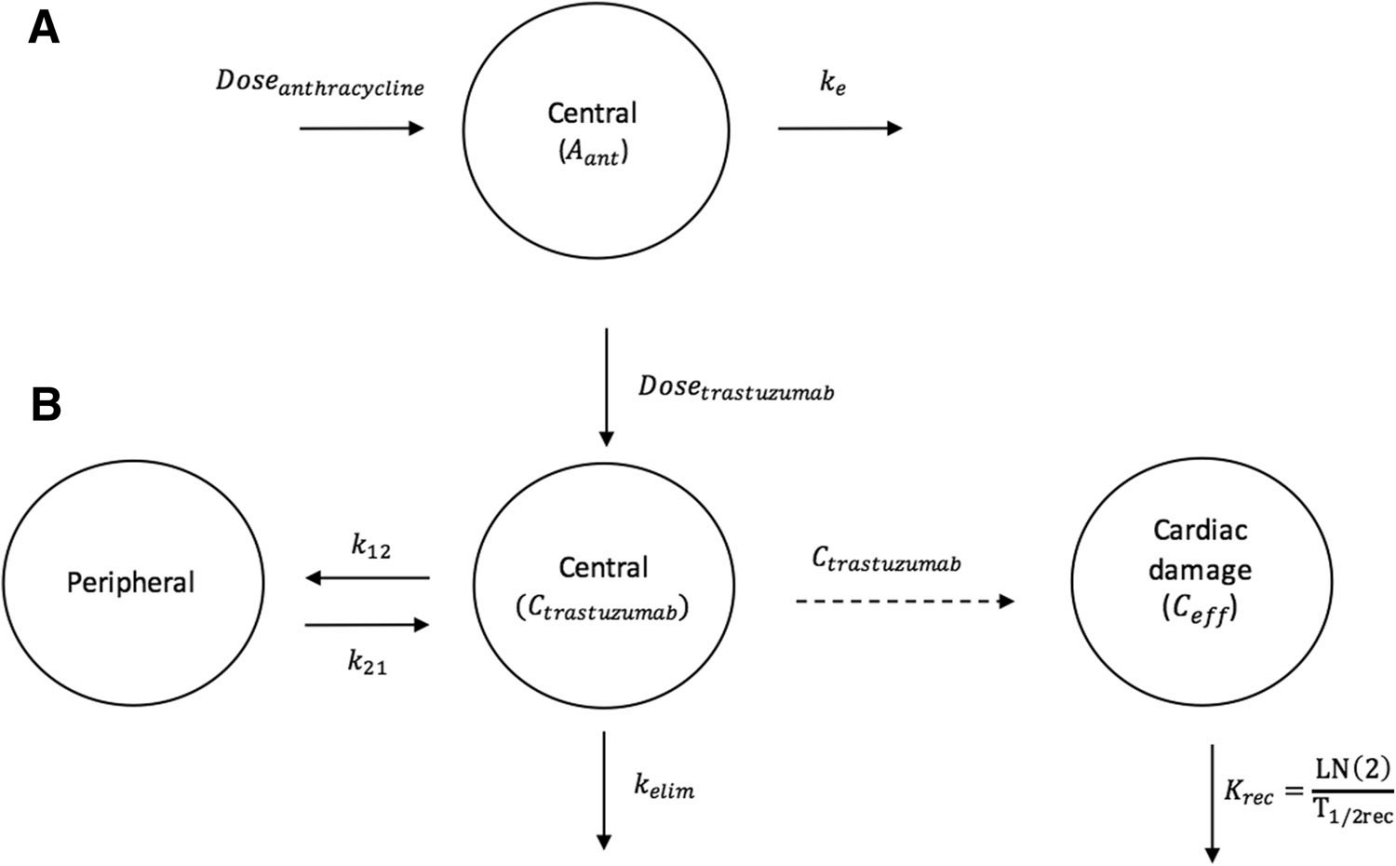
Structural models for anthracycline and troponin T (K-PD) and trastuzumab and LVEF (PK-PD), *K*_*e*_ = elimination rate constant, *A*_*ant*_ = amount of anthracyclines, *C*_*eff*_ = the concentration in the effect compartment, *K*_*rec*_ = recovery rate constant, *T*_1/2*rec*_ = recovery half-life

**Fig. 2:**
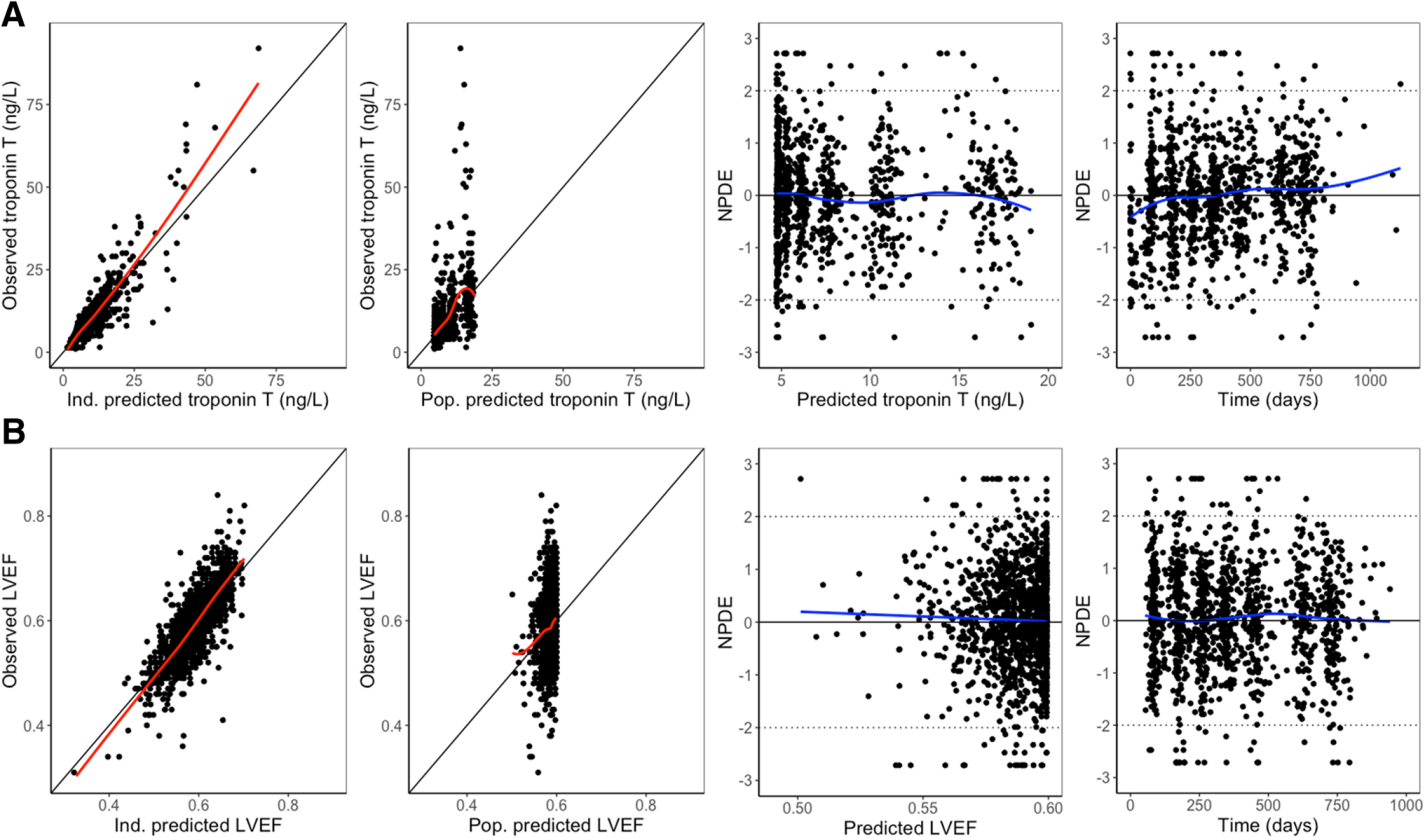
Diagnostic plots for **a** troponin T and **b** left ventricular ejection fractions (LVEF), including individual and population predictions and normalized prediction distribution error (NPDE) over predictions and time

**Fig. 3:**
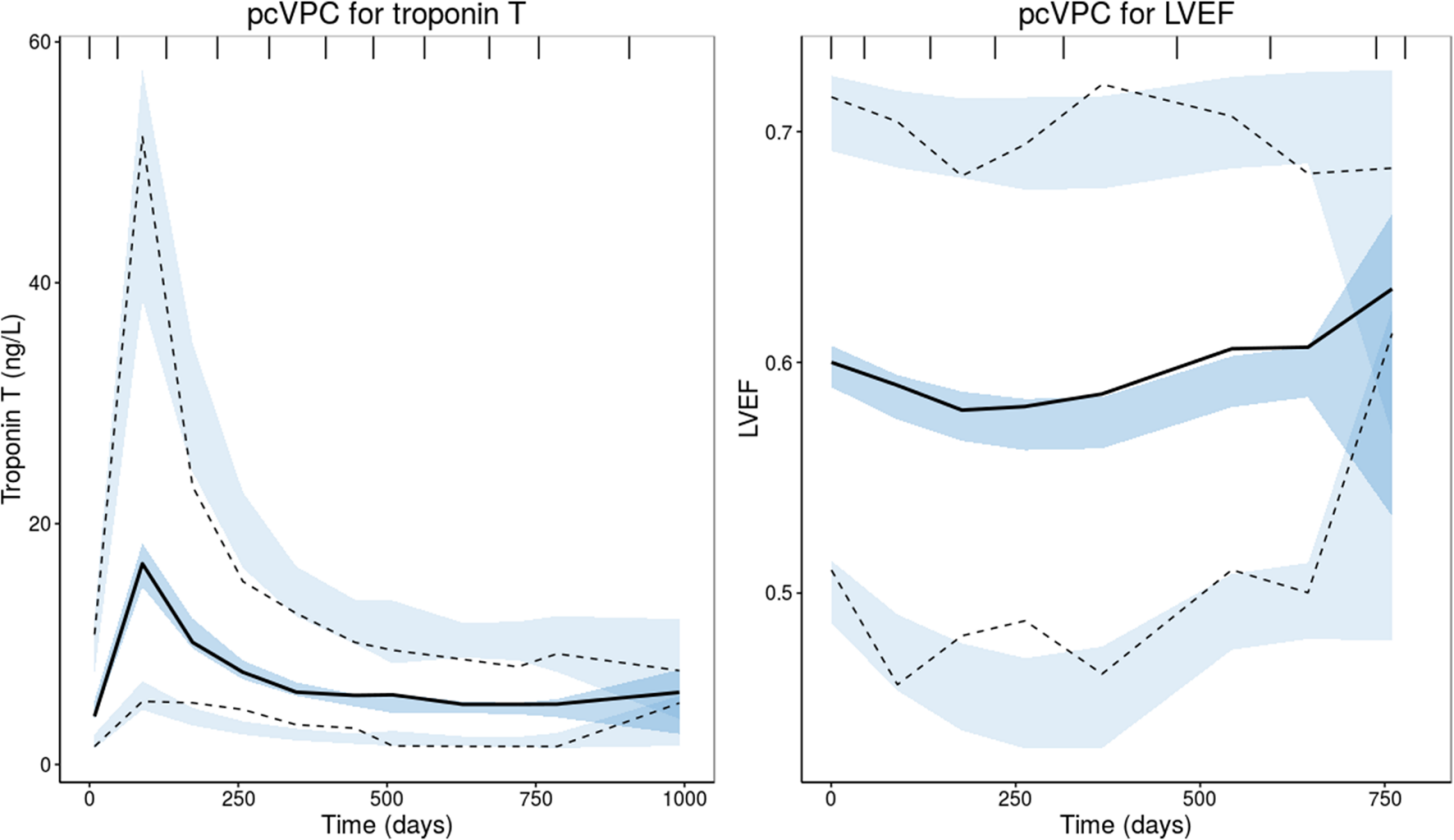
Prediction corrected visual predictive checks (pcVPCs) for troponin T and left ventricular ejection fractions (LVEF). The solid line represents the median of the observed data, the dashed lines represent the 5th and 95th percentiles of the observed data, the shaded areas represent the 95% confidence interval of the simulated data for the corresponding percentiles (n = 500)

Troponin T changes were best described by a direct effect model, where the troponin T concentration increased proportionally with the increment of simulated anthracycline concentrations (Fig. 1a). The model was described by the following equations:

$$\frac{{dA_{ant} }}{dt} = - K_{e} \cdot A_{ant}$$

$$TRP = TRP_{0} \cdot (1 + SLOPE \cdot A_{ant} )$$

where *A*_*ant*_ is the amount of anthracyclines, *K*_*e*_ the elimination rate constant, *TRP* is troponin T, *TRP*_0_ is troponin T at baseline, before starting anthracycline treatment, *SLOPE* is the parameter that describes the proportional increase of troponin T from baseline. The goodness of fit plots (Fig. 2a and Online Resource 1 Fig. S1) showed that the anthracycline-troponin T underpredicted some of the observed higher concentration of troponin T. However, the individual predictions were considered adequate. The VPC showed a slight overprediction of the declining troponin T concentrations in the 95th percentile (Fig. 3), nevertheless, the higher concentrations are described adequately. NPDE plots did not show significant trends. In addition, an indirect effect model was evaluated. However, this model underpredicted the observed concentrations around day 21 post last anthracycline dose and also underpredicted the recovery rate of the troponin T peak.

### LVEF

**Fig. 4:**
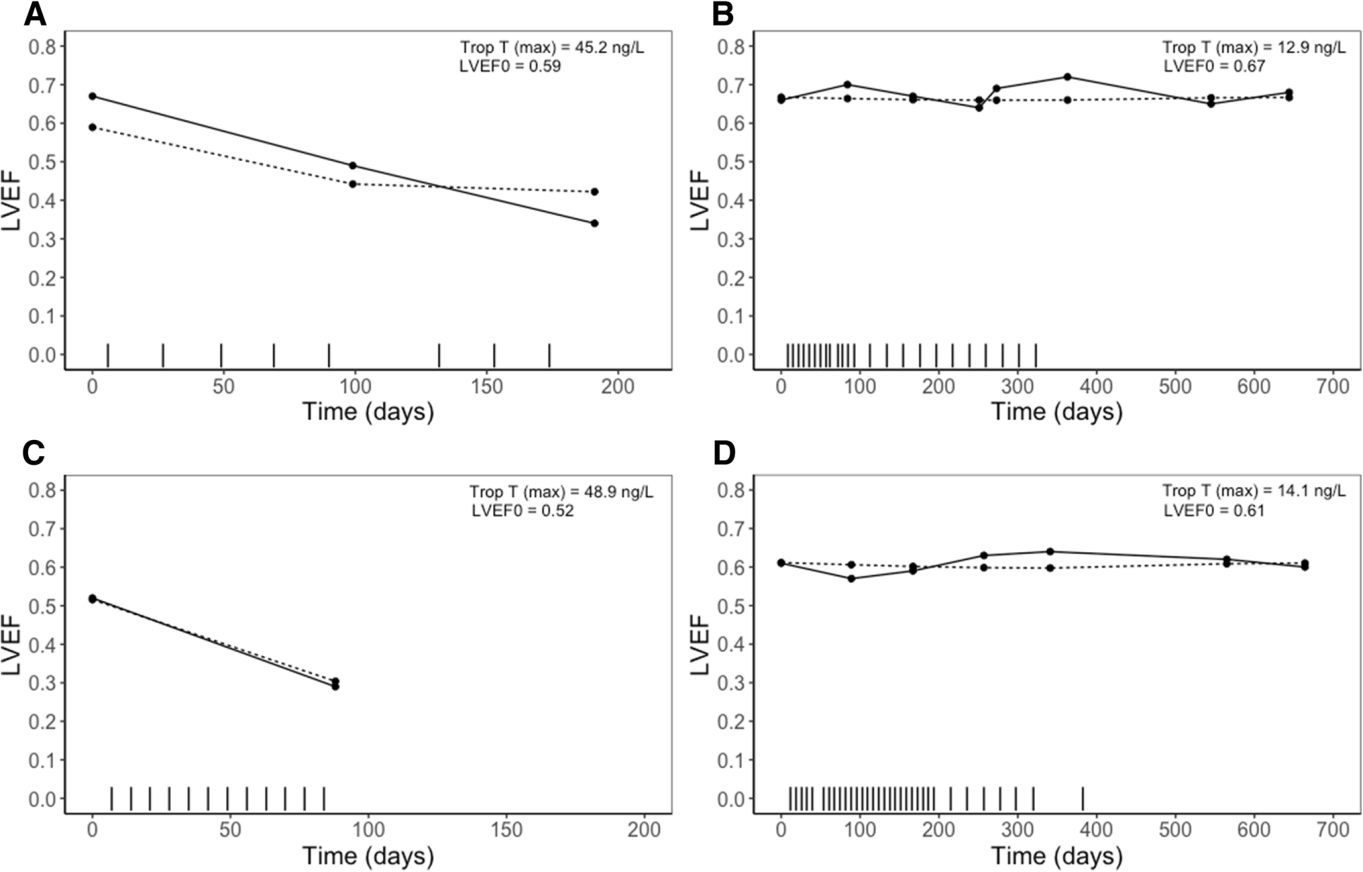
Panels of individual plots for observed (solid line) and individual predicted (dotted line) left ventricular ejection fractions (LVEFs) over time for four different patients. Vertical dashes represent the trastuzumab administrations. **a** Patient with maximum troponin T values in the higher range, relatively high baseline and a significant decline in LVEF value. **b** Patient with lower maximum concentration of troponin T, normal baseline and no significant decline in LVEF. **c** Patient with maximum troponin T values in the higher range, relatively low LVEF baseline and a significant decline in LVEF. **d** Patient with maximum troponin T values in the lower range, relatively high LVEF baseline and no significant decline in LVEF

The previously published LVEF model, used as a modeling starting point, described the data well [19]. However, different models were evaluated to separate the delay in cardiac damage after trastuzumab administration and the recovery rate. In the final model, the cardiac damage was generated by cumulative trastuzumab concentrations and the recovery half-life was estimated. The structural model is depicted in Fig. 1b. The model was described by the following equations:

$$\frac{{dC_{eff} }}{dt} = C_{trastuzumab} - \left( {\frac{\ln \left( 2 \right)}{{T_{1/2rec} }}} \right) \cdot C_{eff}$$

$$LVEF = LVEF_{0} \cdot \left( {1 - \frac{{C_{eff} }}{{C_{eff} + EC_{50} }}} \right)$$

where *C*_*eff*_ is the effect compartment concentration, *C*_*trastuzumab*_ the trastuzumab concentration in the central compartment, *T*_1/2*rec*_ the recovery half-life (where ${ \ln }(2)/T_{1/2rec}$ represents the recovery rate constant), LVEF_0_ the LVEF baseline value before the first administration of trastuzumab and EC_50_ the concentration at which 50% of the drug effect occurs. LVEF decline recovered after cessation of trastuzumab treatment with a recovery half-life of 68 days (RSE 17.2%). The BSV for both EC_50_ and recovery could not be identified, therefore the BSV on recovery was not included in the final model. The BSV in baseline LVEF was moderately positively correlated with the EC_50_ parameter (*r* = 0.585), indicating that patients with a low baseline LVEF tend to have a higher sensitivity to trastuzumab-induced LVEF decline (lower EC_50_). The model described the data adequately, however a slight underprediction was seen for the lower LVEF values (Fig. 2B, Fig. 3 and Online Resource 1—Fig. S1). This is expected to be the result of discontinuation of treatment in patients experiencing a significant decrease in LVEF, for whom follow up LVEF measurements were not available (e.g. Fig. 4a, c). Therefore, the recovery to baseline in these patients was not supported by observations, but was predicted by the model. A decrease in LVEF was classified as significant if LVEF dropped below 45% or when a decrease of 15% from baseline occurred. The final model was evaluated for the candesartan and placebo group separately. No mayor differences were found in magnitude of parameter estimates or model fit, as expected (data not shown).

### NT-proBNP

NT-proBNP did not demonstrate a clear trend over time during anthracycline or trastuzumab treatment and NT-proBNP concentrations did not increase before LVEF decline occurred and did not behave as an inverse of LVEF decline. Therefore, the NT-proBNP baseline concentrations were evaluated as covariates in the LVEF model. These models were also evaluated for each treatment group separately (candesartan and placebo), leading to similar results. However, high relative increases of NT-proBNP, approximately 27 weeks after the lowest LVEF value, were observed in 4 patients with a significant decline in LVEF and in 3 patients with no significant decline in LVEF (Online Resource 1, Figs. S2 and S3). NT-proBNP increment could, therefore, be a delayed effect of previous LVEF decline, a sign of other underlying cardiac disease or a forecast of development of congestive heart failure, since these patients did not report an increase in heart failure symptoms (NYHA) at the time of the peak. Therefore, NT-proBNP was not included in the LVEF model.

### Covariates

The covariates were evaluated on the *SLOPE* parameter (anthracycline-troponin T model) and on the EC_50_ and recovery half-life parameter (trastuzumab-LVEF model). No covariates were identified that significantly improved model fit of the anthracycline-troponin T model, except for type of anthracycline on the *SLOPE* parameter. The *SLOPE*-parameter, was significantly affected by the type of anthracycline administered, as illustrated by twofold lower estimate for epirubicin compared to doxorubicin (anthracycline-type effect estimate of 0.524 (relative standard error (RSE) 17.5%). This means that, for example, administration of one dose of 100 mg doxorubicine leads to an increase in troponin T from 3 to 5.6 ng/L, where an equivalent dose of epirubicin would increase troponin T from 3 to 4.3 ng/L. Considering the LVEF-trastuzumab model, the sensitivity for LVEF decline (EC_50_ parameter) was significantly affected by the predicted maximum concentration of troponin T after anthracycline treatment. Sensitivity for LVEF decline was higher (decreased EC_50_ parameter) for patients with a high maximum concentration of troponin T, resulting in a more pronounced decline in LVEF during trastuzumab treatment (Fig. 4), described by the following equation:

$$EC_{50i} = EC_{50pop} \cdot \left( {\frac{{TRP_{\hbox{max} } }}{18}} \right)^{ - 1.16}$$

where $TRP_{\hbox{max} }$ is the peak concentration of troponin T. According to this equation, a peak concentration of 31 ng/L troponin T, would increase the sensitivity to LVEF decrease by a twofold (twofold decrease in EC_50_). The maximum troponin T concentration reduced the between subject variability in sensitivity for LVEF decline from 98.0% in the base model (Online Resource 1, Table S1) to 82.9% in the covariate model. The sensitivity analysis demonstrated that the predicted concentration of troponin T at 21 days after the last anthracycline dose was an equally significant covariate on the EC_50_ parameter (Online Resource 1, Table S1). The other tested covariates did not significantly improve the model.

## Discussion

The pharmacodynamics of troponin T and LVEF changes during anthracycline and trastuzumab treatment, respectively, were successfully described by the reported models. The maximum concentration of troponin T was a significant determinant of sensitivity to trastuzumab-induced cardiotoxicity, defined as a decline in LVEF values.

Baseline troponin T concentrations were directly affected by anthracycline concentrations. The type of anthracycline significantly affected the linear *SLOPE* parameter, showing that epirubicin had an approximately twofold lower proportional effect on baseline troponin T concentrations compared to doxorubicin. This finding is expected, since at equivalent doses, epirubicin demonstrates less cardiotoxicity than doxorubicin. Moreover, the cumulative lifetime anthracycline dose threshold, associated with increased incidence of heart failure, is also almost a twofold higher for epirubicin (950 mg/m^2^) compared to doxorubicin (550 mg/m^2^) [9, 10]. The anthracycline-troponin T model predicted maximum troponin T concentration at the day of the last anthracycline infusion. However most of the troponin T samples were drawn at approximately 21 days after the last anthracycline dose. To delay the troponin T peak to 21 days, a turnover model was evaluated. However, the recovery rate of troponin T was estimated to be slower than observed, indicating that the peak of troponin T occurs earlier after administration. This is supported by literature, reporting that myocyte damage induced by anthracyclines occurs within hours after administration [25]. In addition, an increase of troponin T within 1–3 days after doxorubicin infusion has been reported in the pediatric setting and an increase in troponin I within hours after high-dose chemotherapy has been reported for adults [26, 27].

The trastuzumab-LVEF model demonstrated a decline in LVEF values that improved after treatment cessation, demonstrated by a recovery of LVEF towards baseline. This analysis prospectively validated the model for LVEF developed in a previously published PK-PD analysis [19]. A high variability in susceptibility to LVEF decline and development of congestive heart failure has been reported in various clinical trials for patients treated with anthracyclines and trastuzumab. High peak troponin T levels were proved to be predictive for this high sensitivity towards trastuzumab induced LVEF decline. The sensitivity analysis demonstrated that the concentration of troponin T at 21 days after the last anthracycline dose is predictive of sensitivity to trastuzumab-induced cardiotoxicity. In addition, the baseline value of LVEF showed to be moderately positive related to the EC_50_ parameter, indicating that patients with a low LVEF baseline tend to be more sensitive to trastuzumab-induced LVEF decrease.

Repeated NT-proBNP measurements were not integrated in the model, since NT-proBNP showed high variability. However, some patients experienced high relative increases of NT-proBNP, approximately 27 weeks after their nadir of the LVEF value. An increment in NT-proBNP could therefore possibly be a delayed effect of a prior decline in LVEF, which could be a sign of other cardiac comorbidities or a forecast of development of congestive heart failure. However, early time course data for NT-proBNP, during anthracycline treatment, was lacking for most patients, which could be a reason why NT-proBNP was not identified as an early cardiac biomarker in this cohort. Additionally, NT-proBNP baseline concentrations were not significantly related to anthracycline-induced troponin T increase nor to LVEF decline during trastuzumab treatment.

Age, cardiac history and interval between anthracycline treatment and initiation of trastuzumab treatment have been previously reported as risk factors for trastuzumab-induced cardiotoxicity [28]. In addition, age and pre-existing cardiac disease have been related to anthracycline-induced cardiotoxicity [29]. In this analysis none of these factors could be identified as covariates influencing either trastuzumab-induced cardiotoxicity or anthracycline-induced cardiotoxicity. This could be related to in- and exclusion criteria of the study, since only patients with favorable cardiac history and relatively low age were included, with a maximum age of 69 years and only 13% of patients being older than 60 years. In addition, for only 6% of patients the length of the interval between end of anthracycline and initiation of trastuzumab treatment was longer than 30 days, explaining why time between treatments could not be identified as a covariate in this analysis. Patients in this study were randomized to receive candesartan or placebo, initiated on the first day of trastuzumab treatment. Therefore, we do not expect that the randomization affects the anthracycline-troponin T model. Candesartan showed no cardio-protective effects in the original study. Nevertheless, to evaluate potential differences between candesartan and placebo for the trastuzumab-LVEF model, treatment group was evaluated as a covariate on the EC_50_ and found not significantly different. In addition, no differences in parameter estimates or model fit were seen when the trastuzumab-LVEF model was evaluated for each treatment group separately.

The biomarker models were not estimated simultaneously. However, anthracyclines induce troponin T release by damaging cardiac cells, where trastuzumab is hypothesized to cause functional changes in contractile proteins, not associated with cell damage or troponin T changes [5–7]. Therefore, we do not expect that the LVEF changes affect the estimation of the anthracycline-troponin T model. In addition, we used the peak troponin T concentration as a covariate, because the amount of troponin T is expected to be a marker for the amount of damage caused by anthracyclines.

The established models can help evaluate the feasibility of using a cardiac biomarker (e.g. troponin T) to identify patients at risk of trastuzumab-induced LVEF decline. However, clinical applicability is challenged by unstandardized analytical assays for determination of cardiac biomarkers and algorithms to calculate LVEF, definition of the optimal sampling time point, identification of a cut-off value and subsequently determination of a proper strategy in case of identification of an abnormal cardiac value. In addition, subclinical LV dysfunction can be estimated using alternative methods, such as determination of LV diastolic dysfunction and myocardial strain. These parameters give insight in early changes in LV remodeling and have been recommended to monitor patients treated with cardio-toxic anti-cancer drugs [30].

Although prospective validation is warranted, the developed models can be applied to evaluate cardiac monitoring strategies, such as previously described for LVEF [12]. Simulation of troponin T and LVEF profiles could aid development of adaptive cardiac monitoring protocols, possibly integrating troponin T as a potential biomarker and determination of optimal sampling time points. Risk-stratified protocols could optimize adaptive dosing in high-risk patients, ensuring maximum possible exposure to trastuzumab, and reduce amount of LVEF measurements in low-risk patients.

In conclusion, to our knowledge this is the first PK-PD analysis that integrated longitudinal data of two cardiac biomarkers during anthracycline and trastuzumab treatment. In this cohort, changes in NT-proBNP could not be demonstrated to be related or predictive of anthracycline- or trastuzumab-induced cardiotoxicity. The analysis identified maximum troponin T concentration after anthracycline treatment as a significant determinant of subsequent trastuzumab-induced LVEF decrease.

## Electronic supplementary material

Below is the link to the electronic supplementary material.

Supplementary material 1 (DOCX 578 kb)
