# Supplementary material for: Pharmacodynamic modeling of cardiac biomarkers in breast cancer patients treated with anthracycline and trastuzumab regimens
Source: J Pharmacokinet Pharmacodyn. 2018 Feb 10;45(3):431–42. doi: 10.1007/s10928-018-9579-8 (PMC5953989; doi:10.1007/s10928-018-9579-8)
Supplement: Supplementary file 1 — Supplementary material 1 (DOCX 578 kb) [file 10928_2018_9579_MOESM1_ESM.docx]

**Online Resource 1**

**Pharmacodynamic modeling of cardiac biomarkers in breast cancer patients treated with anthracycline and trastuzumab regimens.**

Aurelia H.M. de Vries Schultink, Annelies H. Boekhout, Jourik A. Gietema, Artur M. Burylo, Thomas P.C. Dorlo, J.G. Coen van Hasselt, Jan H.M. Schellens, Alwin D.R. Huitema

**Caption** Supplementary plots CWRES plot anthracycline-troponin T model and trastuzumab-LVEF model (Figure S1), for NT-proBNP (Figure S2 and Figure S3) and a table reporting model parameters for the Trastuzumab-LVEF base model and covariate models (peak troponin T and day 21 troponin T) (Table S1)


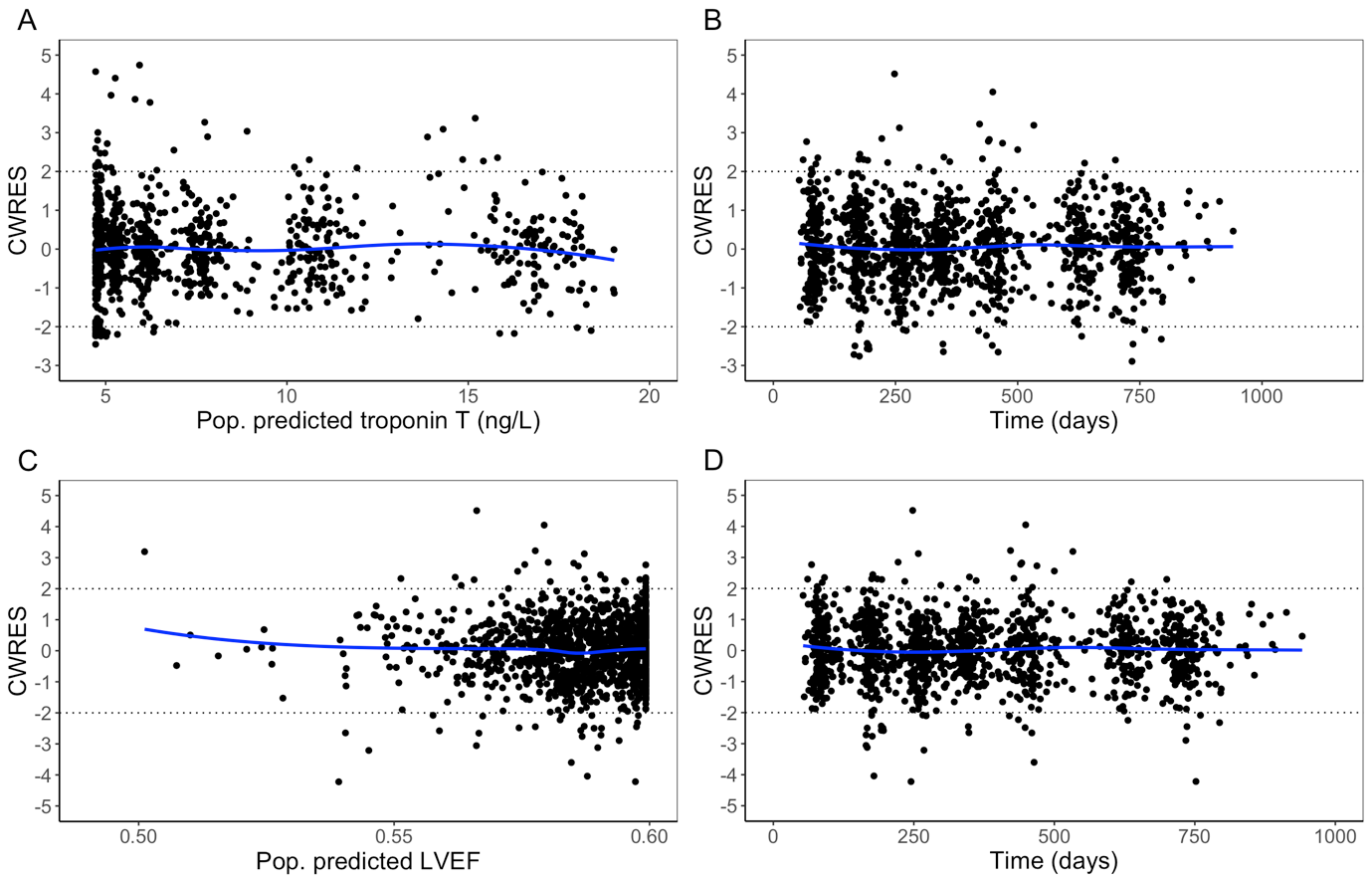


**Figure S1** CWRES plots for the anthracycline-troponin T model (A) CWRES vs. population predictions, (B) CWRES vs. time and for the trastuzumab-LVEF model (C) CWRES vs. population predictions, (D) CWRES vs. time


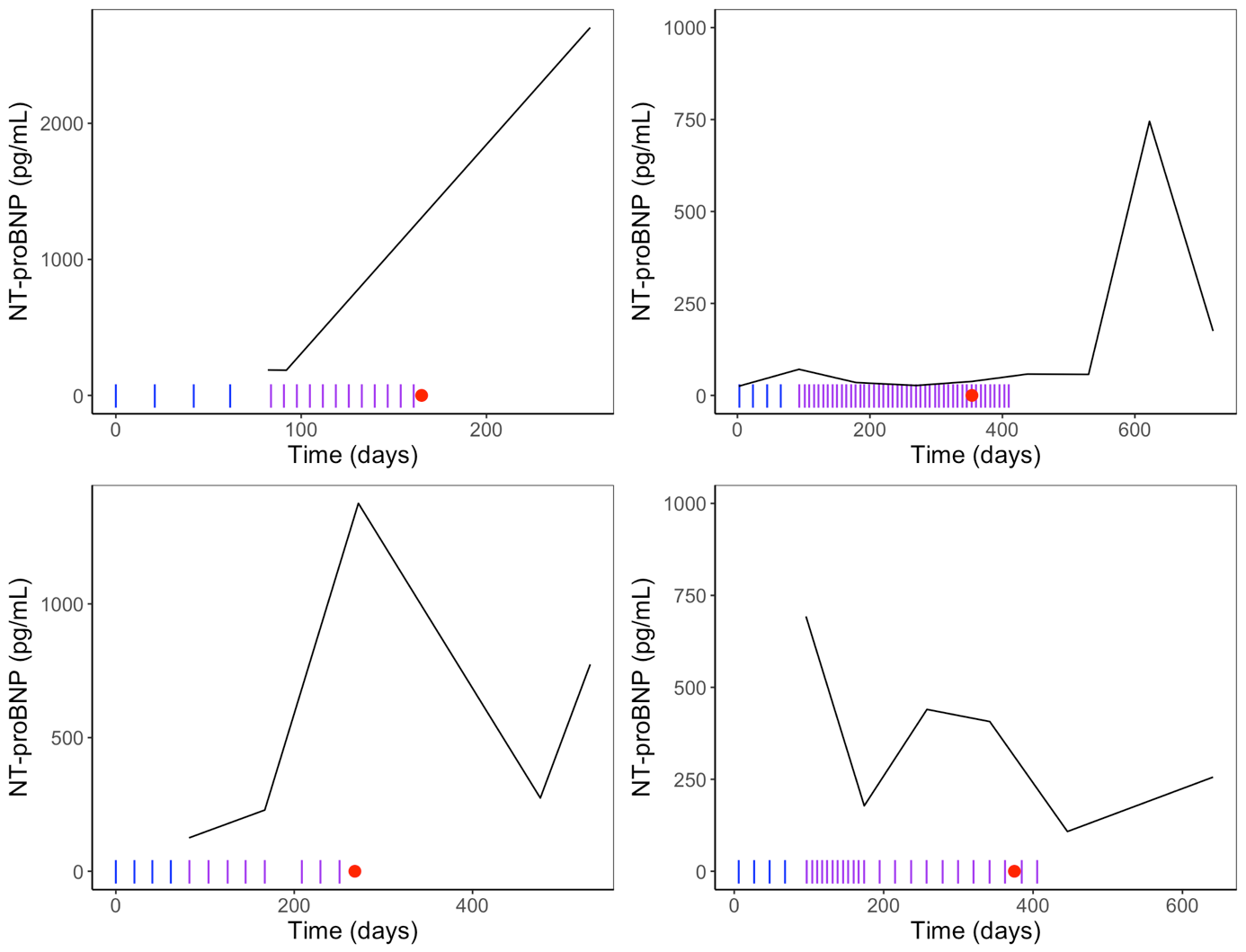


***Figure S2*** *Panels representing individual plots for NT-proBNP changes over time (solid line) for patients that experienced <45% LVEF or 15% decrease of LVEF from baseline. Blue vertical lines represent anthracycline administrations, purple vertical lines represent trastuzumab administrations and the red dot represents the time point at which significant decline of LVEF occurred.*


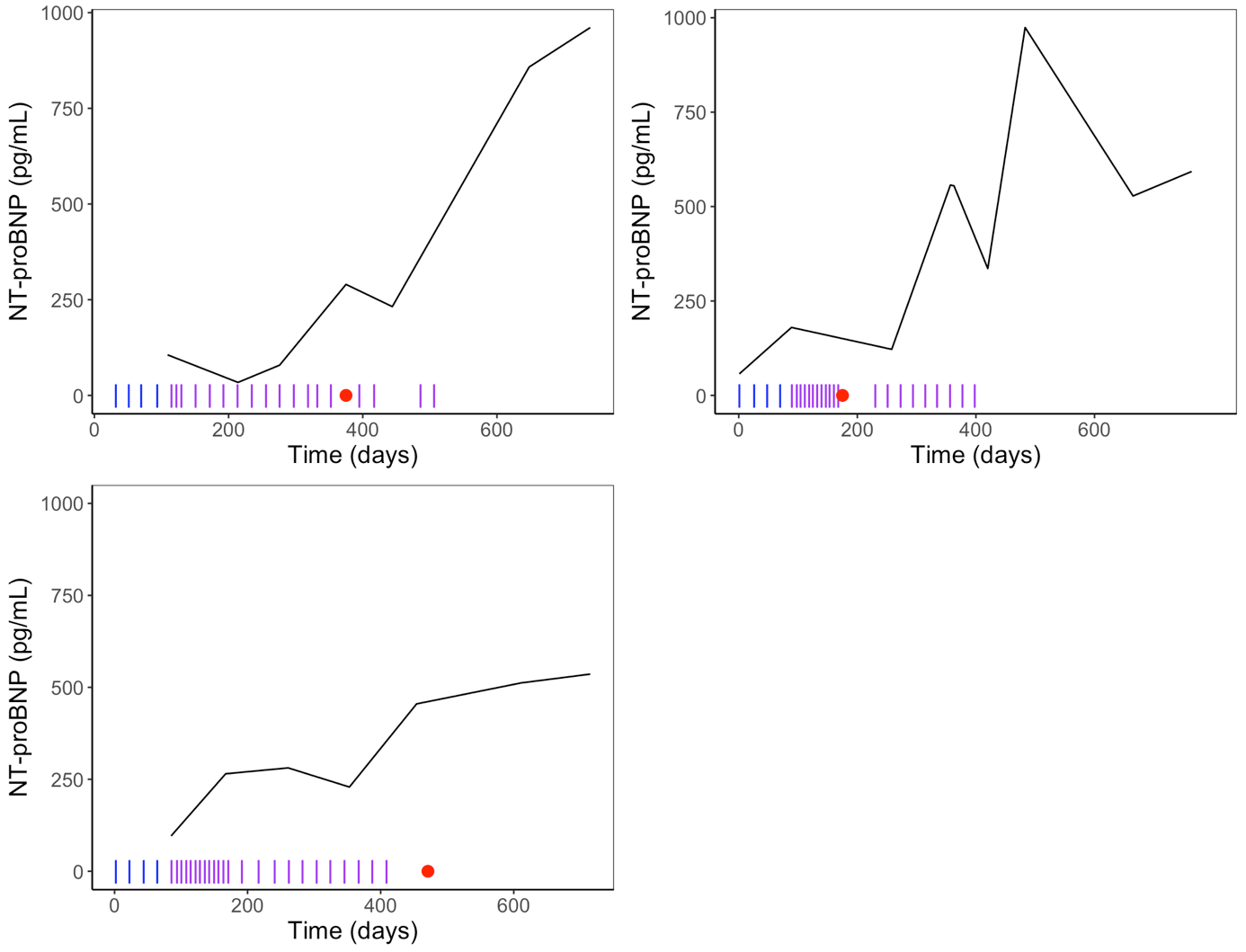


***Figure S3*** *Panels representing individual plots for NT-proBNP changes over time (solid line) for patients that not experienced a significant decline in LVEF, but demonstrated increases in NT-proBNP after treatment. Blue vertical lines represent anthracycline administrations, purple rugs represent trastuzumab administrations and the red dot represents the time point at which the lowest LVEF value occurred.*

**Table S1** Parameter estimates for the trastuzumab-LVEF base model, covariate model with peak concentration of troponin T as covariate, and troponin T concentration at 21 days post the last anthracycline dose.

|  |  | **Base model** | **Troponin T peak conc.** | **Troponin T day 21 conc.** |
| --- | --- | --- | --- | --- |
| Change in objective function value |  | - | -19 | -19 |
| **Parameters** | **Unit** | **Estimate (RSE%) [shrinkage%]** | | |
| LVEF baseline value (LVEF_0_) |  | 0.6 (0.6) | 0.599 (0.6) | 0.599 (0.6) |
| Recovery half-life (REC) | day | 69.6 (16.5) | 67.9 (17.2) | 67.8 (16.1) |
| Sensitivity to LVEF decline (EC_50_) | mg/L | 1.93∙10^5^ | 2.18∙10^5^ (23.4) | 2.19∙10^5^ (23.1) |
| Maximum troponin T effect on EC_50_ |  | - | -1.16 (23.4) | -1.19 (23.1) |
|  |  |  |  |  |
| *Between-subject variability (%)* | | | |  |
| LVEF baseline value (LVEF_0_) | CV | 7.0 (8) [11.1] | 7.07 (16.7) [9.9] | 7.1 (8.0) [9.7] |
| Sensitivity to LVEF decline (EC_50_) | CV | 98.0 (9) [23] | 82.9 (43.1) [26] | 82.3 (17) [26] |
| Correlation $\omega$_LVEF0_~$\omega$_EC50_^a^ | - | 0.568 | 0.585 | 0.584 |
|  |  |  |  |  |
| *Residual variability* | | | |  |
| Proportional residual error LVEF | % | 7.9 (2.8) [9.7] | 7.8 (2.9) [8.3] | 7.8 (2.9) [8.2] |

*CV = coefficient of variation, SD=standard deviation, RSE = relative standard error.*

*^a^Correlation derived from the variance-covariance matrix of the random effects.*
